# Causes of different goiter rates with the same iodine deficiency among the pastoral and agricultural populations of Tibet: a geographical comparison

**DOI:** 10.1186/s13044-022-00122-8

**Published:** 2022-03-19

**Authors:** Jing Xu, Shichuan Liu, Wei Ma, Xiuwei Li, Min Guo, Xiaoxiao Cao, Yunyou Gu, Haiyan Wang, Jianqiang Wang, Ying Zhang, Guangxiu Zhuang, Liejun Liu

**Affiliations:** 1 Chinese Center for Disease Control and Prevention, Key Laboratory of Trace Element Nutrition of National Health Commission of China, National Institute for Nutrition and Health, 155 Changbai Road, Changping, Beijing, 102206 China; 2 Hangzhou Center for Disease Control and Prevention, 568 Mingshi Road, Jianqiao Town, Hangzhou, 310021 Jianggan District China; 3 Tibet Autonomous Region Center for Disease Control and Prevention, 71 Jinzhu Middle Road, Lhasa, 850002 China

**Keywords:** Iodine, Thyroid hormone, Thyroid goiter, Iodine deficiency, Tibet

## Abstract

**Background:**

The residents of both the agricultural and pastoral areas of Tibet share the same iodine deficiency and iodine nutrition, but the rate of thyroid goiter was significantly higher in the agricultural areas than in the pastoral areas. This project sought to determine why the populations in the iodine-deficient pastoral areas show a lower rate of thyroid goiter.

**Methods:**

Food frequency questionnaires (FFQs) and 24 h history recalls were adopted to investigate the dietary patterns of the residents of the agricultural and pastoral areas. Meat and milk samples were collected to measure their inorganic iodine, total iodine and thyroid hormone contents using ICP-MS, As^III^-Ce^4+^ catalytic spectrophotometry and the Siemens’ chemiluminescence method, respectively. The intake of protein, and the microelements, selenium and iron, was calculated according to their content in the food.

**Results:**

The per capita daily intake of meat, dairy, and cereal in the pastoral areas was 116.7, 216.7, and 433.3 g, respectively, which are significantly higher than those in the agricultural areas (50.0, 72.2, and 375.0 g, respectively) (*p* < 0.05). The content of thyroid hormone in dried beef and milk in the pastoral areas was 62.6 and 13.5 μg/kg, respectively, which was significantly higher than those in the agricultural areas (25.1 and 4.1 μg/kg, respectively) (*p* < 0.05). The daily intake of thyroid hormone, protein and microelements, selenium and iron from foods by the residents of the pastoral areas were 10.5 μg, 99.6 g, 30.0 μg and 15.8 mg respectively, which was significantly higher than those in the agricultural areas (1.79 μg, 56.5 g, 23.8 μg and 13.2 mg, respectively) (*p* < 0.05).

**Conclusions:**

The significantly high intake of the food-borne thyroid hormone by the residents of the pastoral area could be the main reason the residents in the pastoral areas show a lower rate of thyroid goiter than those in the agricultural area. Moreover, the relatively high intake of protein and trace elements, selenium and iron by residents in the pastoral area could be another important factor for reducing the goiter rates.

## Introduction

Iodine deficiency may cause thyroid goiter, cretinism, and developmental disorders in the embryonic brain, all of which are collectively referred to as iodine deficiency disorders (IDD) [1]. Normally, IDD prevalence is closely correlated with the level of iodine deficiency in the environment. However, a strange phenomenon is occurring in Tibet, China, where the residents of both the agricultural and pastoral areas of Tibet share the same iodine deficiency, but the IDD prevalence between these populations is significantly different. Prior to 1995, when China had not implemented the universal salt iodization (USI) policy, the median urinary iodine concentration (UIC) of children was 40–50 μg/L in most agricultural and pastoral areas, and less than 20 μg/L in a few areas [2–4], indicating moderate and severe iodine deficiency, respectively, according to the WHO classification standards for IDD [1]. Moreover, the residents in the agricultural areas of Tibet had a significantly higher rate of thyroid goiter (40 – 52%) than those in the pastoral areas (0%). Cretinism was also prevalent in the agricultural areas (2–13%) but was not observed in the pastoral areas (0%) [4]. After the complete implementation of the USI policy, in 1996 [5], iodized salts became widely available in agricultural areas, and the iodine levels in farming residents were fundamentally improved. However, there are a multitude of arid salt lakes in the pastoral areas of Tibet; therefore, natural table salt is free and can be easily acquired by the local herdsmen. Thus, the use of iodized salt among families in the pastoral areas and the iodine levels in these residents have not been improved. Despite this, the thyroid goiter rate in the pastoral areas was still lower than that in the agricultural areas, where residents had an adequate iodine intake (UIC >100 μg/L), after the USI policy implementation. Based on China’s IDD monitoring data from 1985 [4], 2002 [6], 2011 [7], and a study by Dudan et al. in 2009 [8], the median UIC and thyroid goiter rates of children in the pastoral and agricultural areas before and after USI are shown in Table 1. Our previous study, from 2010, found that the protein intake of pastoral residents was significantly higher than that of agricultural residents, which may be an important reason for the low prevalence of goiter in the pastoral areas [9, 10]. However, a high intake of protein alone is not sufficient to account for the reduced rate of thyroid goiter among the residents of the pastoral areas during severe iodine deficiency.

**Table 1:** UIC (μg/L) and thyroid goiter rates of children before and after USI,in 1996

year	UIC (μg/L)		Goiter rate (%)	
	Pastoral area	Agricultural area	Pastoral area	Agricultural area
1985	40.0	55	–^a^	40
2002	42.2	132.9	8.9	13.5
2009	50.2	193.2	2.0	16.0
2011	68.1	187.2	1.5	2.8

^a^No goiter was found on palpation

This project sought to determine the main reason for the lower rate of thyroid goiter in the populations of iodine-deficient pastoral areas.

## Materials and methods

### Field survey and sampling

Naqu county and Chanang county were selected from the pastoral and agricultural areas, respectively, for the field survey. The residents in Naqu county primarily depend on livestock farming for living, and the residents in Chanang county primarily live from crop farming, and their lifestyle and food structure have remained unchanged for years. Both counties are self-sufficient in terms of economy and lifestyle. The geographical and climatic environments of the two counties are described in Table 2 [11].

**Table 2:** Geographical and climatic environment of the pastoral Naqu county and the agricultural Chanang county

	Population	Area (km^2^)	Altitude (m)	Annual mean temperature (℃)	Annual mean precipitation (mm)
Naqu	90,000	16,195	4,450	-2.2	400
Chanang	83,000	2,163	3,620	8.2	420

The sample size was determined using the formula: *n* = t_ɑ_^2^(1 - p) p/d^2^, which is usually adopted to estimate the minimum sample size for cluster sampling or simple random sampling [12]. We considered the average goiter rate (11%) of Tibetan children in 2005 as the probability (p), 1.96 as the t-critical value (t_ɑ_, ɑ = 0.05), and 10% as the allowable sampling error (d). Therefore, the sample size for each area should be 38 participants, at least [*n* = 1.96^2^(1 - 0.11)0.11/0.1^2^ = 37.6].

Two towns each in the pastoral Naqu county and the agricultural Chanang county were selected as the investigation sites for the dietary survey. The selected towns met the following requirements: the populations should be relatively concentrated (more than 2,000 habitants per town), have low mobility, a similar economic level, and stable living habits. One hundred households from each area were randomly sampled, according to the local household registration, and one adult aged 18–55 years was selected from each family to participate in the dietary interview. In total, there were 200 participants, with an approximately equal number of men and women.

Food frequency questionnaires (FFQs) and the 24 h history recall were adopted to assess the dietary habits of the residents over the current and the previous year [13–15], with emphasis on meat and dairy consumption. Based on the dietary habits of Tibetan people, questions were asked about the intake of meat (yak beef, dried yak beef, cattle beef, dried cattle beef, mutton, pork, and chicken), dairy (cow milk, sheep milk, butter, and yogurt), cereals (barley, wheat, and rice), potatoes, vegetables (Chinese cabbage, green pepper, tomato, cauliflower, eggplant, lettuce, radish, mushroom, pumpkin, carrot, and cucumber), and eggs. The response options were defined as follows: “never/rarely,” “1–5 times per six months,” “1–3 times per month,” “1 time per week,” “2–4 times per week,” “5–6 times per week,” “1 time per day,” and “2+ times per day.” Subsequently, the 24 h history recall (24 h recall) was conducted for three consecutive days to collect the dietary data. The seasonal food intake, such as lamb in the winter and autumn, was calculated according to the annual consumption. The daily food intake was estimated using a food map developed by Nanjing Medical University, China [16].

The survey group consisted of eight well-trained surveyors and one experienced epidemiologist. The unified FFQ and 24 h recall questionnaires were used to conduct the household surveys, which were carried out one by one in agricultural areas first, and then in the pastoral areas. The investigators participated together in the first five household surveys for each area, and then conducted separate investigations.

Dried beef, milk, mutton, and pork were sampled and stored in an ice box, and then transported to the laboratory to measure the iodine and thyroid hormone levels.

### Method for measuring the thyroxine levels in milk

The milk samples were centrifuged at 3,000 rpm (*r* = 16 cm) for 10 min, and the intermediate layer was used to measure the thyroxine (T_4_) content using the chemiluminescence method. The analysis was performed using a Siemens Adivia Centaur system and the corresponding kits. The recovery, intra- and inter-assay variation coefficients of this method were 89.2–91.6%, 3.96%, and 5.83% (*n* = 6), respectively [17].

### Method for measuring the thyroxine levels in meat

First, the thyroxine in beef jerky was determined using the method recommended by the American Thyroid Association (ATA), in which thyroxine was extracted using methanol: chloroform (1:2) and its level was determined using radioimmunoassay (RIA) [18]. However, the results exhibited poor repeatability. The intra-assay variation coefficient ranged from 9.3% to 25% among the six random samples. It is possible that the protein denaturation of beef jerky makes the protein-bound thyroxine difficult to extract completely. Therefore, the method of measuring the organic iodine content in dried beef was subsequently adopted to determine the levels of thyroxine. The iodine level in dried beef primarily comes from the blood and comprises both organic and inorganic iodine, and 90% of organic iodine is thyroid hormone [19–22]. Therefore, the total iodine and inorganic iodine levels were measured in dried beef, and their differential values were considered as the levels of organic iodine. There are two types of thyroid hormones, T_3_ and T_4_, with the latter being more prevalent. Therefore, the T_4_ levels are used to indicate the levels of thyroid hormone in dried beef and can be calculated based on the organic iodine (I_o_) content, according to the following formula (1):

1 $${\mathrm{T}}_{4}\left(\mu \mathrm{g}/k\mathrm{g}\right)=\mathrm{Io}/\left(4\times 127\right)\times 777\times 90\%$$

Where:

4: one T_4_ molecule contains 4 iodine atoms;

127: iodine relative atomic weight;

777: T_4_ relative molecular weight;

90%: 90% of organic iodine is thyroid hormone in the tissues.

To extract the inorganic iodine from dried beef, a 0.4 g sample was placed in a 5 mL centrifuge tube, to which 3 mL of deionized water and two stainless steel or ceramic grinding beads with a diameter of 2 mm were added. Next, a pipe cover was installed, and the apparatus was placed into a vertical vibrating ball grinder and ground for 120 s at a frequency of 65 Hz. The centrifuge tube and its contents were then centrifuged for 5 min at 4,000 rpm. The supernatant was removed, the process was repeated three times, and the supernatants were pooled at the end. Deionized water was added to the supernatant to reach a final volume of 10 mL. Next, 1.0 mL of 20% (w/w) trichloroacetic acid solution was added to the supernatants for precipitation of the protein, and the samples were placed into a constant-temperature water bath at 37 °C for 30 min, followed by centrifugation at 3,500 rpm for 10 min. The thyroid hormone combined with protein and the dissociative thyroid hormone (heavier than water and insoluble in water) settled at the bottom of the tube, separate from the inorganic iodine. Then, 4.0 mL of the supernatant was carefully extracted and filtered with a 5-μm syringe aqueous-phase filter membrane to measure the inorganic iodine content.

The extracted inorganic iodine was measured using ICP-MS [23]. The recovery rate was used to evaluate the effects of the extraction of inorganic iodine from the samples. A total of 1 mL of an iodine standard solution was added to 0.4 g of dried beef paste to reach an iodine content of 50 μg/L in the supernatants in the end. The recovery was calculated using the following formula:

$$Recovery\mathrm{ \% }= (inorganic iodine after standard iodine was added- inorganic iodine in the sample)/(standard iodine added)\times 100\mathrm{\%}$$

The recovery and intra-assay variation coefficients (cv%) of the method for extracting and measuring the inorganic iodine level in dried beef were 93.8 ± 2.3% with a range of 89.7–96.8% and 2.6% (2.4% to 2.9%), respectively.

The total iodine levels in dried beef were measured using As^III^–Ce^4+^ catalytic spectrophotometry and dry ash sampling, and the detection limit of this method was 6 μg/kg for a 0.5 g sample [24]. The reference material 8418 (wheat gluten, NIST of USA) with iodine content of 60±13 μg/kg was used to control the quality of the test. The ICP-MS method is not suitable for the determination of total iodine in dried beef because of the high inorganic salt content of samples after ashing.

### Data input and statistical analysis

The collected data were sorted and summarized. Excel software was used to establish a database and the statistical analyses were performed using SPSS20.0. The D test (R. B. D’ Agostino) was used as a normality test to determine the type of distribution of the quantitative data. For skewed distribution data, the median values were adopted to describe the centralized trend and indicate the mean levels. The interquartile range was used to determine the degree of dispersion. The Wilcoxon signed-rank test was adopted to determine the significance of the differences between two groups of samples, and a value of *p* < 0.05 was accepted as statistically significant. For the normal distribution data, the mean values and standard deviations were adopted to describe the mean levels and the degree of dispersion. The statistical t-test was adopted for significance assessment, and a value of *p* < 0.05 was accepted as statistically significant.

## Results

### Participants

The number of male and female participants was approximately the same, and the age structure was not significantly different between the two areas.

### Resident food structure and primary food intake

In the pastoral area, the food structure is predominantly composed of meat, dairy, grain, and cereal foods. Meats primarily included yak beef, dried yak beef, and lamb, and no pork or poultry. Dairy foods primarily included yak milk, milk tea, and yogurt made from yak milk. In the agricultural area, the food structure primarily included grains, cereals, and vegetable food, with meats and dairy foods comprising a smaller portion of the diet. Meats primarily included cattle beef, dried cattle beef, lamb, and a small quantity of pork and chicken. Dairy products primarily included cattle milk and their products. The beef, mutton, cow milk, and goat milk consumed by residents of the agricultural and pastoral areas were primarily self-supplied. The beef was mostly air-dried and transformed into dried beef for long-term preservation (approximately 2.35 kg of fresh beef is needed to generate 1 kg of dried beef). A small quantity of fresh beef was frozen for long-term preservation. Both areas had similar grain, cereal, and vegetable food varieties. Grain and cereal foods primarily included barley, wheat, and rice. The vegetables primarily included Chinese cabbage, radish, green pepper, and cucumber. The vegetables in the pastoral areas are primarily supplied by the agricultural areas. Both areas are inhabited by Tibetans, who do not have the habit of eating fish or seafood.

**Fig. 1:**
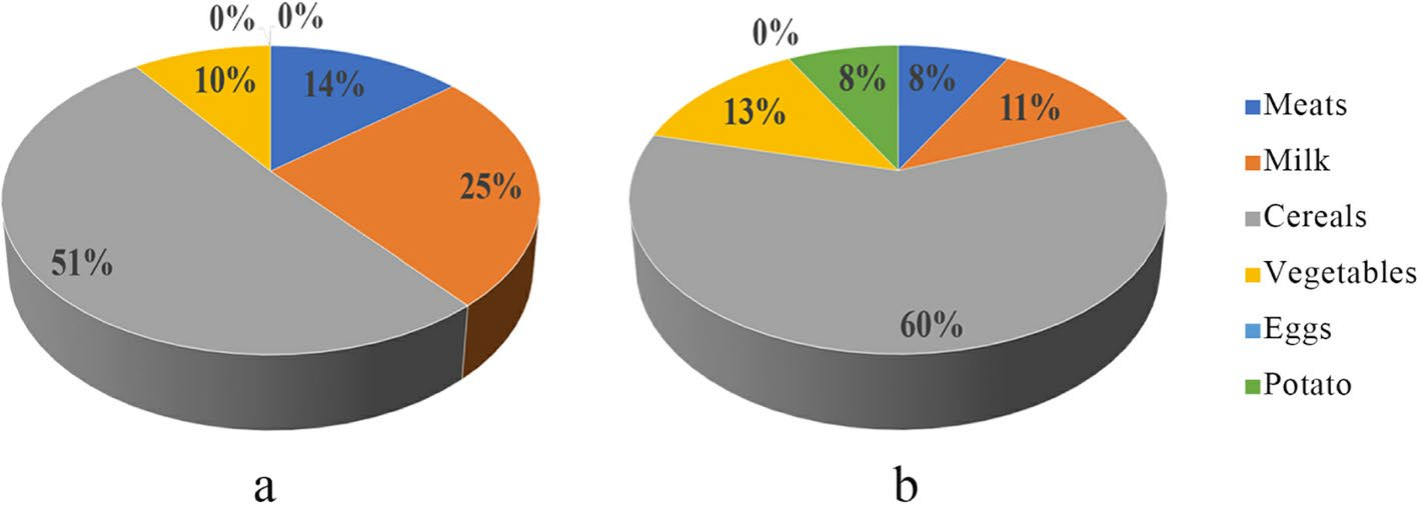
The daily food composition of residents in pastoral areas (**a**) and agriculture areas (**b**)

After food categorization, the daily intake of meats, dairy, cereals, vegetables, potatoes, and eggs per capita was compiled as shown in Table 3. The quantity of fresh beef was proportionately converted to the quantity of dried beef (Table 3). The intake of meat, dairy, and cereal foods was significantly higher in the pastoral areas than in the agricultural areas (*p* < 0.05). The potato consumption was higher in the agricultural areas than in the pastoral areas (*p* < 0.05). The consumption of vegetables and eggs was not significantly different between the two areas (*p* > 0.05). The daily food consumption of the residents of the pastoral and agricultural areas is shown in Figure 1 a and b, respectively.

**Table 3:** Daily intake of foods (g) per capita in the pastoral and agricultural areas

	Pastoral residents’ daily intake			p	Agricultural residents’ daily intake		
	Median	Interquartile	Range		Median	Interquartile	Range
Dried beef	80.9	37.6	11.6-323.7	0.000	46.2	41.8	8.9-155.6
Mutton	83.3	41.7	33.3-100.0	0.232	25.0	85.4	16.7-125.0
Pork	16.7	16.7	0.0-33.3	0.233	33.3	33.3	8.3-133.3
**Meat total**	**116.7**	**66.7**	**0.0-420.0**	**0.000**	**50.0**	**47.9**	**0.0-166.7**
Milk	216.7	475.0	0.0-1250.0	0.000	72.2	202.1	0.0-1000.0
Eggs	0.0	0.0	0.0-1.0	0.614	0.0	0.0	0.0-1.33
Barley	100.0	50.0	41.7-266.7	0.649	133.3	85.4	0.0-416.7
Wheat	183.3	91.7	16.7-366.7	0.222	166.7	83.3	16.7-400.0
Rice	133.3	83.3	33.3-300.0	0.000	83.3	66.7	8.3-333.3
**Cereal total**	**433.3**	**125.0**	**150-700**	**0.000**	**375.0**	**108.3**	**150-666.6**
Chin. Cabbage	50.0	33.3	16.7-150.0	0.009	66.7	50.0	16.7-183.3
Green pepper	33.3	16.7	16.7-100.0	0.156	16.7	16.7	16.7-50.0
Tomato	16.7	8.3	16.7-33.3	0.016	50.0	50.0	33.3-100.0
Cauliflower	16.7	0.0	0.0-16.7	0.022	33.3	12.5	16.7-50.0
Eggplant	16.7	16.7	16.7-33.3	-	0.0	0.0	0.0
Lettuce	33.3	16.7	16.7-100.0	0.072	33.3	16.7	16.7-66.7
Radish	33.3	20.8	16.7-66.7	0.288	33.3	33.3	16.7-83.3
Mushroom	16.7	12.5	16.7-83.3	0.104	33.3	25.0	16.7-66.7
Pumpkin	16.7	37.5	16.7-100.0	0.817	25.0	4.5	16.7-33.3
Cucumber	16.7	16.7	16.7-66.7	0.957	25.0	4.5	16.7-33.3
**Vegetable total**	**83.3**	**62.5**	**0-300**	**0.155**	**83.3**	**66.7**	**0-250**
Potato	0.0	16.6	0-133.3	0.000	50	83.3	0-216.6

### Resident protein intake

Protein intake was calculated by multiplying the food intake by the protein content in the foods. The protein content in foods (primarily meat, dairy, and cereals with a high protein content) was sourced from the Chinese Food Composition [25]. The calculated protein intake of residents is shown in Table 4. The per capita daily protein intake was 99.6 g in the pastoral area, which was higher than the intake of 80 g/day recommended by the Chinese Nutrition Society for an adult male who performs moderate physical labor [25]. In contrast, the protein intake was 56.5 g in the agricultural area, which was far lower than the recommended intake.

**Table 4:** Per capita daily intake of protein (g) in the pastoral and agricultural areas

	Pastoral residents’ daily intake			p	Agricultural residents’ daily intake		
	Median	Interquartile	Range		Median	Interquartile	Range
Meat protein	53.2	38.0	0-212.8	0.000	15.2	30.4	0-76.0
Dairy protein	8.0	8.8	0-25.3	0.000	4.4	1.7	0-15.4
Cereal protein	41.8	12.2	11.7-69.0	0.006	36.0	13.2	12.1-65.2
Total	99.6	43.1	17.8-293.3	0.000	56.5	27.3	12.5-140.3

### Thyroid hormone levels in meat and milk

Ninety-five dried yak beef samples and 100 yak milk samples were collected from families in the pastoral areas. In total, 75 dried cattle beef samples, 10 pork samples, and 100 cattle milk samples were collected from families in the agricultural areas. The investigation took place in the summer, and the residents seldom consumed mutton and sheep milk in the summer. Only one mutton sample was collected from an agricultural area. The test results for iodine and thyroid hormone levels in the meat and dairy foods are shown in Table 5. The results revealed that the levels of thyroid hormone were significantly (2.5 times) higher in the dried beef samples from the pastoral areas than those from the agricultural areas (*p* < 0.05). The level of thyroid hormone in cow milk was also significantly (3.3 times) higher in the pastoral areas than in the agricultural areas (*p* < 0.05). Such differences may primarily arise from the different species of cattle used in these areas. The pork samples from the agricultural areas contained almost no thyroid hormone.

**Table 5:** Iodine and thyroxine levels in meat and dairy foods (μg/kg)

*N*^a^=97, 75, 100, 10 and 1	Pastoral area			p	Agricultural area		
	Median	Interquartile	Range		Median	Interquartile	Range
Total iodine in dried beef	100.8	53.0	39.7-287.8	0.000	52.5	37.4	10.4-382.5
Inorganic iodine in dried beef	55.8	27.3	11.9-166.5	0.001	33.7	25.7	1.1-104.0
Organic iodine in dried beef	45.5	39.4	6.8-245.6	0.000	18.2	22.7	0.4-278.5
thyroxine in dried beef	62.6	54.2	9.4-338.1	0.000	25.1	31.2	0.6-383.4
thyroxine in milk	13.5	7.0	0.0-83.2	0.000	4.1	6.6	0.0-117.2
thyroxine in mutton	-	-	-	-	10.1	-	-
thyroxine in pork	-	-	-	-	0.2	0.3	0.0-0.9

^a^97 dried beef samples from pastoral area, 75 dried beef samples from agricultural area, 100 milk samples from each area, 10 pork samples and 1 mutton sample from agricultural area

In order to observe the stability of the thyroid hormone in beef jerky, six samples with low (11.01 μg/kg, 14.04 μg/kg), medium (61.26 μg/kg, 65.11 μg/kg) and high (171.4 μg/kg, 215.3 μg/kg) levels of thyroid hormone were selected and assessed again after being stored at 4 °C for 3 months. The average relative deviation between the two measurements was -5.4% with a range of -13.2%–2.2%, which indicated that the thyroid hormone in beef jerky was stable.

Six beef jerky samples were initially measured using the method recommended by the ATA for the determination of thyroid hormone in the tissues [18]. Although the reproducibility of the ATA method for measuring thyroid hormone in beef jerky was poor, there was a good correlation (*r* = 0.8445)between the mean values measured using the ATA method and those measured using the organic iodine method. The content of thyroid hormone in beef jerky determined using the organic iodine method was higher of 20% than that determined using the ATA method.

### Intake of thyroid hormone from meat and dairy sources by the residents

The content of thyroid hormone in the meat and milk of each household was multiplied by the average amount of meat and milk consumed by each household member to obtain the per capita daily intake of the thyroid hormone, as shown in Table 6. The per capita daily intake of thyroid hormone was significantly (6 times) higher in the pastoral areas than in the agricultural areas (*p* < 0.05).

**Table 6:** Per capita daily intake (μg) of thyroid hormone from meat and dairy

Source of thyroid hormone	Pastoral residents’ daily intake of thyroid hormone			p	Agricultural residents’ daily intake of thyroid hormone		
	Median	Interquartile	Range		Median	Interquartile	Range
Beef	4.98	4.65	0.86-38.8	0.000	1.09	1.52	0.02-22.2
Milk	3.74	7.80	0.14-40.6	0.000	0.93	4.14	0.0-74.3
Beef and milk	9.62	10.8	0.90-47.2	0.000	1.54	2.95	0.06-75.3
mutton	0.84	-	-	-	0.25		
Total	10.5				1.79		

### Intake of selenium and iron microelements by the residents

Selenium and iron alleviate thyroid goiter [26–29]. To obtain the per capita daily intake of selenium and iron in the pastoral and agricultural areas, the residents’ daily intake of various foods in the agricultural and pastoral areas was multiplied by the mean levels of selenium and iron in the foods. The mean levels of selenium and iron in the various foods were sourced from the "Chinese Food Composition" (2004) [25]. Table 7 shows that the intake values of selenium and iron were both higher in the pastoral areas than in the agricultural areas, and this difference was statistically significant (*p* < 0.05).

**Table 7:** Per capita daily intake of selenium and iron

*N* =100	Selenium intake (μg)			Iron intake (mg)		
	Median	Interquartile	Range	Median	Interquartile	Range
Pastoral residents	30.0	10.3	7.0~77.4	15.8	5.8	7.2~37.1
Agricultural residents	23.8	10.3	6.9~58.8	13.2	6.4	4.5~32.2
p	0.000			0.001		

## Discussion

### Effects of iodine, thyroid hormone, and protein on the thyroid function

Since the implementation of USI in 1996, the rate of goiter of the residents in the agricultural areas decreased from 40% to 2.8% in 2011 and 1.5% in 2017. while the rate of goiter in the pastoral area has remained at a low level (0.0% in 1985, 1.5% in 2011 and 1.4% in 2017) [4, 7, 30]. The results of dietary survey showed that the types of grains and vegetables in agricultural and pastoral areas were basically the same, and only potato intake in agricultural areas was higher than that in pastoral areas. Therefore, iodine deficiency is believed as the main cause of goiter prevalence in agricultural areas before USI, and some natural goitrogens, which may exist in grain and vegetables, contributed little to the prevalence of goiter.

Iodine has biological effects only when it contributes towards the synthesis of thyroid hormones within the body. However, not all iodine obtained from the diet can be utilized for thyroid hormone synthesis in the human body and some of it is excreted through the kidneys. In people who have a low protein intake, such as vegetarians, the bioavailability of iodine is limited [31–34]. The bioavailability of thyroid hormones is approximately 100%, and almost all thyroid hormones absorbed from food can enter the bloodstream to directly perform their biological functions [35, 36]. As a result, consuming iodine in the form of thyroid hormone is more effective than taking inorganic iodine to compensate for the lack of thyroid hormone synthesis arising from iodine deficiency. The results of this survey show that the dietary intake of thyroid hormone of the residents in the pastoral areas was significantly higher than that in the agricultural areas, which may be the main reason for the significantly lower goiter rate of the residents in the pastoral areas, compared to that in the agricultural areas. The experimental results showed that the thyroid hormone in dried beef had good stability. Even if deiodinase is present in beef, it may be denatured and deactivated in dried beef or the enzymatic reaction may not occur under dry conditions. Clinically, the recommended dose of thyroxine for treating hypothyroidism was initially 35 μg per day for children and 50 μg per day for pregnant women with subclinical hypothyroidism [37, 38]. This treatment regimen was specifically designed for patients with hypothyroidism due to thyroid dysfunction. However, the residents in the pastoral areas were different from patients with thyroid dysfunction, with their thyroid glands being normally enriched in iodine to synthesize thyroid hormones. The thyroid hormone synthesized by the pastoral residents and the thyroid hormone ingested from meat and milk food (although it was only 10.5 μg per day) could still maintain the normal levels of thyroid function, sparing them from the iodine deficiency disorders. Moreover, the relatively high intake of protein and trace elements selenium and iron by residents in the pastoral area, which effectively promotes the absorption and use of iodine in the body, could be another important factor for reducing the goiter rates.

We did not directly measure the iron and selenium content in the residents’ food in the two regions, and the intake of iron and selenium calculated from the "Chinese Food Composition" may be a little rough.

### Reasons for the elevated thyroid hormone levels in meat and yak milk from the pastoral areas

The pastoral areas are at a high altitude (above 4,000 m), with very low temperatures and low amounts of oxygen. Herdsmen have lived on pasturage for generations, primarily by raising yaks. To adapt to the blistering cold environment, yaks may have developed an increased ability to store nutrients. The literature indicates that yak beef and milk have higher levels of minerals, proteins, and iodine than those from other types of bovines [39]. A report on the increased iodine levels in yak beef and milk compared to cattle beef and milk matches the conclusion of this study. The water iodine levels in the pastoral areas (median 1.3 µg/L) are higher than those in the agricultural areas (median 0.7 µg/L) [10], potentially promoting favorable conditions for yaks to gather and reserve iodine for the synthesis of thyroid hormone. In addition to the inborn quality of yaks in the pastoral areas, the slaughter via drowning instead of exsanguination and yak beef production through natural drying, rather than water washing, also increases the blood content in the muscles, contributing to the higher levels of thyroid hormone in dried yak beef.

## Conclusions

The significantly high intake of the food-borne thyroid hormone by residents in the pastoral area, which effectively compensates for the lack of synthetic thyroid hormones arising from iodine deficiency in the human body, could be the main reason residents in the pastoral areas show a lower rate of thyroid goiter than those in the agricultural area. Moreover, the relatively high intake of protein and trace elements, selenium and iron by residents in the pastoral area, which effectively promotes the absorption and use of iodine in the body, could be another important factor for reducing the goiter rates.

## Data Availability

The data described in the manuscript, code book, and analytic code will be made available upon request.
